# Metformin and Ara-a Effectively Suppress Brain Cancer by Targeting Cancer Stem/Progenitor Cells

**DOI:** 10.3389/fnins.2015.00442

**Published:** 2015-11-23

**Authors:** Tarek H. Mouhieddine, Amaly Nokkari, Muhieddine M. Itani, Farah Chamaa, Hisham Bahmad, Alissar Monzer, Rabih El-Merahbi, Georges Daoud, Assaad Eid, Firas H. Kobeissy, Wassim Abou-Kheir

**Affiliations:** ^1^Department of Anatomy, Cell Biology and Physiological Sciences, Faculty of Medicine, American University of BeirutBeirut, Lebanon; ^2^Department of Biochemistry and Molecular Genetics, Faculty of Medicine, American University of BeirutBeirut, Lebanon

**Keywords:** glioblastoma, neuroblastoma, metformin, Ara-A, AMPK pathway, cancer stem cell

## Abstract

**Background:** Gliomas and neuroblastomas pose a great health burden worldwide with a poor and moderate prognosis, respectively. Many studies have tried to find effective treatments for these primary malignant brain tumors. Of interest, the AMP-activated protein kinase (AMPK) pathway was found to be associated with tumorigenesis and tumor survival, leading to many studies on AMPK drugs, especially Metformin, and their potential role as anti-cancer treatments. Cancer stem cells (CSCs) are a small population of slowly-dividing, treatment-resistant, undifferentiated cancer cells that are being discovered in a multitude of cancers. They are thought to be responsible for replenishing the tumor with highly proliferative cells and increasing the risk of recurrence.

**Methods:** Metformin and 9-β-d-Arabinofuranosyl Adenine (Ara-a) were used to study the role of the AMPK pathway *in vitro* on U251 (glioblastoma) and SH-SY5Y (neuroblastoma) cell lines.

**Results:** We found that both drugs are able to decrease the survival of U251 and SH-SY5Y cell lines in a 2D as well as a 3D culture model. Metformin and Ara-a significantly decreased the invasive ability of these cancer cell lines. Treatment with these drugs decreased the sphere-forming units (SFU) of U251 cells, with Ara-a being more efficient, signifying the extinction of the CSC population. However, if treatment is withdrawn before all SFUs are extinguished, the CSCs regain some of their sphere-forming capabilities in the case of Metformin but not Ara-a treatment.

**Conclusion:** Metformin and Ara-a have proved to be effective in the treatment of glioblastomas and neuroblastomas, *in vitro*, by targeting their cancer stem/progenitor cell population, which prevents recurrence.

## Introduction

Primary malignant tumors of the brain and central nervous system (CNS) are estimated to have a yearly incidence rate of about 7.25 per 100,000 in the United States (Ostrom et al., 2014). Gliomas are among the most common and detrimental brain cancers with a mean survival time of about 1.5 years (Chang et al., 2006). On the other hand, neuroblastoma is the most common extra-cranial solid tumor in the pediatric age group, with a survival rate ranging between 30 and 90% (Schmidt et al., 2000; Gatta et al., 2002; Maris, 2010). To this date, the treatment for gliomas and neuroblastoma consists of total surgical resection, radiotherapy and chemotherapy (Sathornsumetee et al., 2007; Irwin and Park, 2015). Many studies have utilized different approaches to target various elements contributing to the development of these malignancies.

One element that is gaining increasing interest in cancer treatment is the cancer stem cell (CSC) population found within the primary tumor bulk. CSCs are believed to be the reason behind the initiation, growth, metastasis, and recurrence of tumors as they exhibit self-renewal, differentiation, and tumorigenic properties (Visvader, 2011; Magee et al., 2012). The hallmark of CSC viability is their ability to withstand the potency of conventional therapy, specifically via multi-drug resistance rendering the need for a more targeted approach for therapy (Abdullah and Chow, 2013). The existence of CSCs has been documented in many types of cancers, including gliomas and neuroblastomas (Walton et al., 2004; Chen et al., 2012).

Metformin, usually used as a first line treatment for Type II Diabetes Mellitus, has shown significant promises in treating different types of cancer (Najbauer et al., 2014; Deng et al., 2015; Fujihara et al., 2015). The interest in using Metformin in cancer research arose when several epidemiological studies revealed a lower incidence of cancer in diabetic patients on metformin therapy as compared to patients who are not (Hwang et al., 2015). Metformin, an adenosine monophosphate-activated protein kinase (AMPK) activator, was effective in suppressing and killing glioma and neuroblastoma cells (Isakovic et al., 2007; Shackelford and Shaw, 2009; Kumar et al., 2014; Liu et al., 2014). Furthermore, Metformin proved that it could target the CSC population in glioblastoma tumors (Wurth et al., 2013), rendering it a good adjuvant chemotherapeutic agent. On the other hand, the AMPK inhibitor, 9-β-d-Arabinofuranosyl Adenine (Ara-a), has been mostly used as an inhibitor of Metformin's effect (Takatani et al., 2011), and studies focused on its derivatives in having effective anti-cancerous properties in hematologic malignancies (White et al., 1982; Bogdahn et al., 1987a,b; Lozano-Santos et al., 2015; Sharma et al., 2015c; Valdez et al., 2015).

In this study, we investigated the potency of Metformin and Ara-a in suppressing the proliferation, viability, and invasive potential of the glioblastoma and neuroblastoma cell lines U251 and SH-SY5Y, respectively. We further focused on the effect of these drugs on the CSC population among these cell lines within a 3D model, by using the sphere formation assay within Matrigel™ (Abou-Kheir et al., 2010, 2011; El-Merahbi et al., 2014). We studied the effect of these drugs over five consecutive generations of sphere-formation to determine the potency of abolishing the CSC population.

## Materials and methods

### Cell culture and treatments

U251 and SH-SY5Y cells (ATCC, USA) were cultured and maintained in Dubelcco's Modified Eagle Media (DMEM) Ham's F-12 (Sigma-Aldrich) supplemented with 10% heat inactivated fetal bovine serum (FBS) (Sigma-Aldrich) and 1% Penicillin/Streptomycin (Sigma-Aldrich). Cells were incubated at 37°C in a humidified incubator containing 5% CO_2_. The drugs Metformin and Ara-a were purchased from Sigma-Aldrich and were both reconstituted in dimethyl sulfoxide (DMSO).

### MTT/cell viability assay

The anti-proliferative effects of Metformin and Ara-a were measured *in vitro* by using MTT [(3-(4, 5-dimethylthiazol-2-yl)-2, 5-diphenyltetrazolium bromide)] assay according to the manufacturer's instructions (Roche). Briefly, cells were seeded (1 × 10^4^ cells/well) in 100 μl complete medium in three different 96-well plates—one plate per time point (24, 48, 72 h)—and incubated overnight at 37°C, 5% CO_2_ before being exposed to the different treatments. At each time point, media was removed and replaced with fresh media along with 10 μl/well of the MTT yellow dye and incubated for 4 h, after which 100 μl/well of the solubilizing agent was added and incubated overnight at 37°C, 5% CO_2_. Absorbance intensity was measured by the microplate ELISA reader (Multiscan EX) at 595 nm. The percentage of cell viability was presented as an optical density (OD) ratio of the treated to the untreated cells.

### Wound healing assay

SH-SY5Y and U251 cells were cultured in six-well plates (5 × 10^5^ cells/well) and incubated at 37°C, 5% CO_2_ until they reached 90–100% confluence. Cells were then treated with 10 mg/ml of Mitomycin C (Sigma) for 2 h in order to block cellular proliferation. A sterile 200 μl tip was used to create scratch wounds of the same width on each monolayer. The plates were then washed twice with phosphate-buffered saline (PBS) to remove the detached cells, and the remaining cells were cultured in complete media with or without treatment. Photos were taken at 0, 24, and 48 h, and the distance traveled by the cells enumerated the closure of the wounds.

### Trans-well invasion assay

SH-SY5Y and U251 cells were seeded in the top chamber of Matrigel™-coated inserts (pore size: 8 μm; Falcon) placed in 24-well plates (2 × 10^5^cells/well), while a medium supplemented with 10% FBS was used as a chemo-attractant in the lower chamber. The wells were coated with 100 ml of Matrigel™ (BD Bioscience) at a dilution of 1:10 in cold PBS and air-dried overnight in a biosafety cabinet. The cells were allowed to invade through the Matrigel™ for 24 h at 37°C in a 5% CO_2_ incubator. Cells that did not invade were scraped off with a cotton-tip applicator while the invading cells were fixed and stained with Hematoxylin and Eosin. The number of invading cells was counted under a light microscope (x10 objective) from six consecutive fields for each well.

### 3D culture and sphere-formation assay

Single SH-SY5Y and U251 cell suspension were suspended in Matrigel™/serum free DMEM (1:1) at a concentration of 10^4^cells/well in a total volume of 50 μl. The solution was then plated gently around the rim of individual wells of a 24-well plate and allowed to solidify for 1 h at 37°C in a humidified incubator containing 5% CO_2_. 0.5 ml of DMEM with 2% FBS (for U251) or 5% FBS (for SH-SY5Y) was added gently to the center of each well and the media (containing the treatment) was changed every 2–3 days. Spheres were counted and/or harvested at day 9 (for U251) or day 14 (for SH-SY5Y) after plating. For sphere propagation, the medium was aspirated and the Matrigel™ was digested with 0.5 ml Dispase solution (Invitrogen, Carlsbad, CA, 1 mg/ml, dissolved in serum-free DMEM Ham's F-12) for 60 min at 37°C. Spheres were collected, incubated in 1 ml warm Trypsin- EDTA at 37°C for 5 min, and then passed through a 27-gauge syringe five times. Cells were counted by a hemocytometer and re-seeded at 10^4^cells/well. The sphere-forming unit (SFU), expressed as %, was calculated by dividing the number of spheres counted by the number of input cells (10^4^ cells) and then multiplied by 100. Zeiss Axiovert microscope was used for the acquisition of bright field images.

### Zymography

SH-SY5Y and U251 cells were cultured in petri dishes and incubated at 37°C, 5% CO_2_. The cells were then treated for 24 h, after which the media was collected in falcon conical tubes and stored at −80°C. Protein quantification was done using the Detergent Compatible (DC) Protein Assay (Bio-Rad) as per manufacturer's recommendations, employing bovine serum albumin as a standard. Finally, equal amount of proteins were re-suspended in sample buffer in the absence of reducing agents and loaded onto 7% polyacrylamide gels impregnated with gelatin (3 mg/ml). Protein-containing culture supernatant was then loaded in sodium dodecyl sulfate-polyacrylamide gel (100 μg/well), subjected to electrophoresis and run at low temperature (apparatus placed on ice). Gels were then washed twice at room temperature for 30 min in a 2.5% Triton X-100 solution in IX running buffer. After that, gels were incubated for 24 h in substrate buffer (50 mM Tris-HCl, 5 mM CaCl_2_, 0.02% NaN_3_, pH 8.0) at 37°C, and stained for 2 h, at room temperature, in 0.05% Coomassie blue R-250, in 50% methanol, and 10% acetic acid. Gel destaining with water took place overnight and gelatinases were visualized as clear white bands reflecting digestion of the gel by matrix metalloproteinase (MMP). MMP-2 intensity was calculated by measuring the band size via Image J software.

### Data analysis

Statistical analysis was performed using Statistical Package for Social Sciences program (SPSS) version 20. The significance of the data was analyzed using a Student's *t*-test, and *p*-values of *p* < 0.05 (^*^) and *p* < 0.01 (^**^) were considered significant and highly significant, respectively.

## Results

### Metformin and Ara-a inhibit U251 and SH-SY5Y cell proliferation in a dose- and time-dependent manner

**Figure 1:**
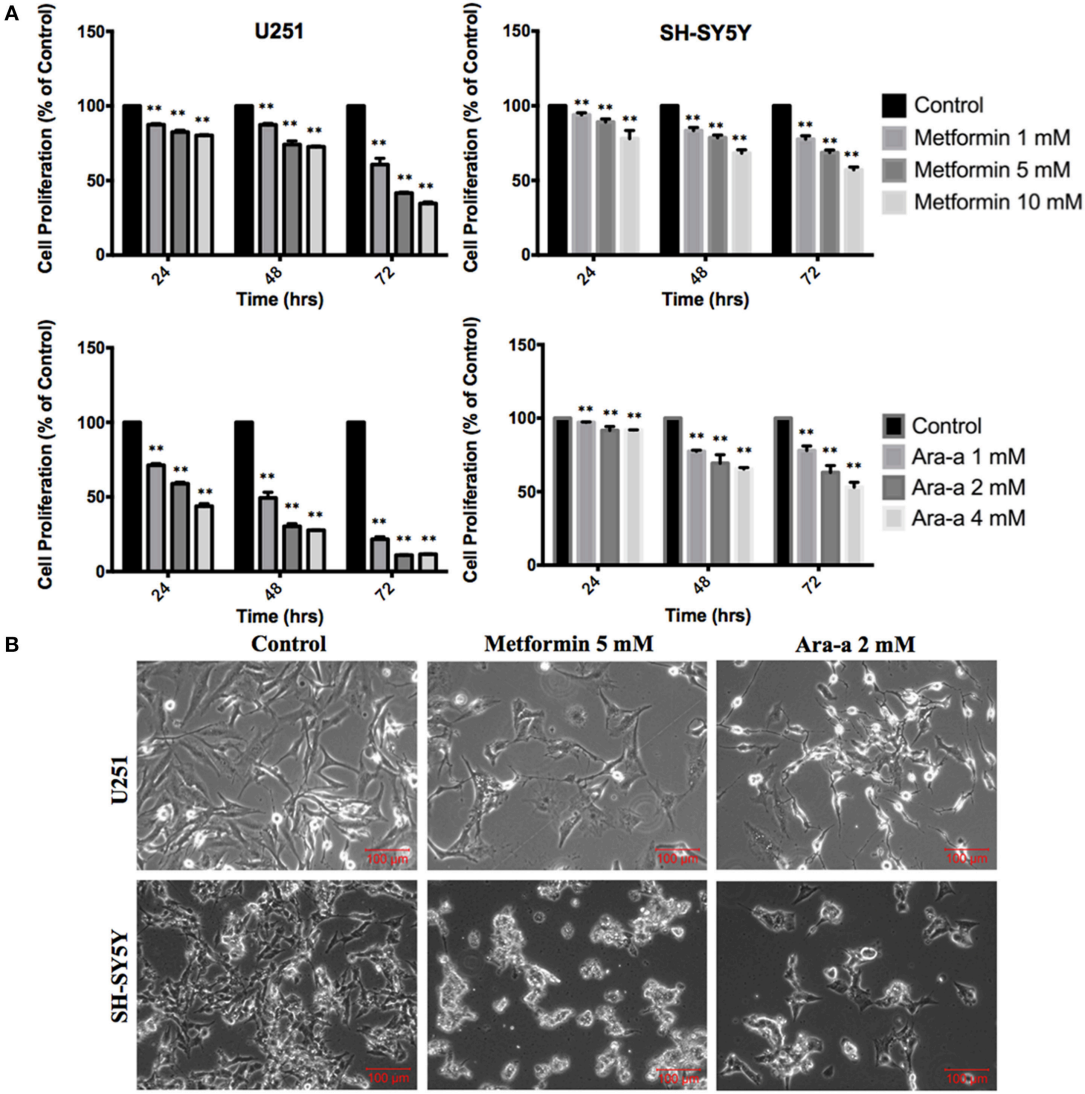
**The effect of Metformin and Ara-a on the proliferation, morphological, and confluence changes of U251 and SH-SY5Y cell lines. (A)** After incubation of the two cell lines (U251 and SH-SY5Y) for 24, 48, and 72 h with or without treatment with Metformin or Ara-a at different concentrations, cell proliferation was determined using MTT assay. Results are expressed as a percentage of the treated group compared to its control. Data represent an average of three independent experiments. The data are reported as mean ± SEM (^**^P < 0.01). **(B)** Representative images of U251 and SH-SY5Y cells were taken after 48 h in culture with or without Metformin and Ara-a treatment. Cells were visualized by Axiovert inverted microscope from Zeiss at 4x magnification.

We first studied the *in vitro* effect of Metformin and Ara-a on the cell proliferation of both cell lines, via MTT assays (Figure 1A). Knowing that the SH-SY5Y cell line has both adherent and floating cells, the media, including floating cells, was removed and thus the MTT results are only reflective of the adherent cells. We found that Metformin similarly decreased the proliferative activity of both U251 and SH-SY5Y cell lines, reaching an inhibitory effect of around 50% at 72 h at 5 mM for U251 and at 10 mM for SH-SY5Y. On the other hand, Ara-a revealed a more potent effect and at a lower concentration, compared to Metformin. There was a 50% inhibitory effect on SH-SY5Y proliferation at 72 h at 4 mM, while the same inhibitory effect was achieved on U251 cells after 24 and 48 h of 2 mM and 1 mM of Ara-a treatment, respectively. Furthermore, we noticed that in addition to inhibiting cell proliferation, Metformin and Ara-a induced cell death in both cell lines, as measured by trypan blue exclusion method (data not shown). The morphological and confluency changes following drug treatment in both cell lines after 48 h are shown in Figure 1B.

### Metformin and Ara-a inhibit the migratory and invasive ability of cancer cell lines

**Figure 2:**
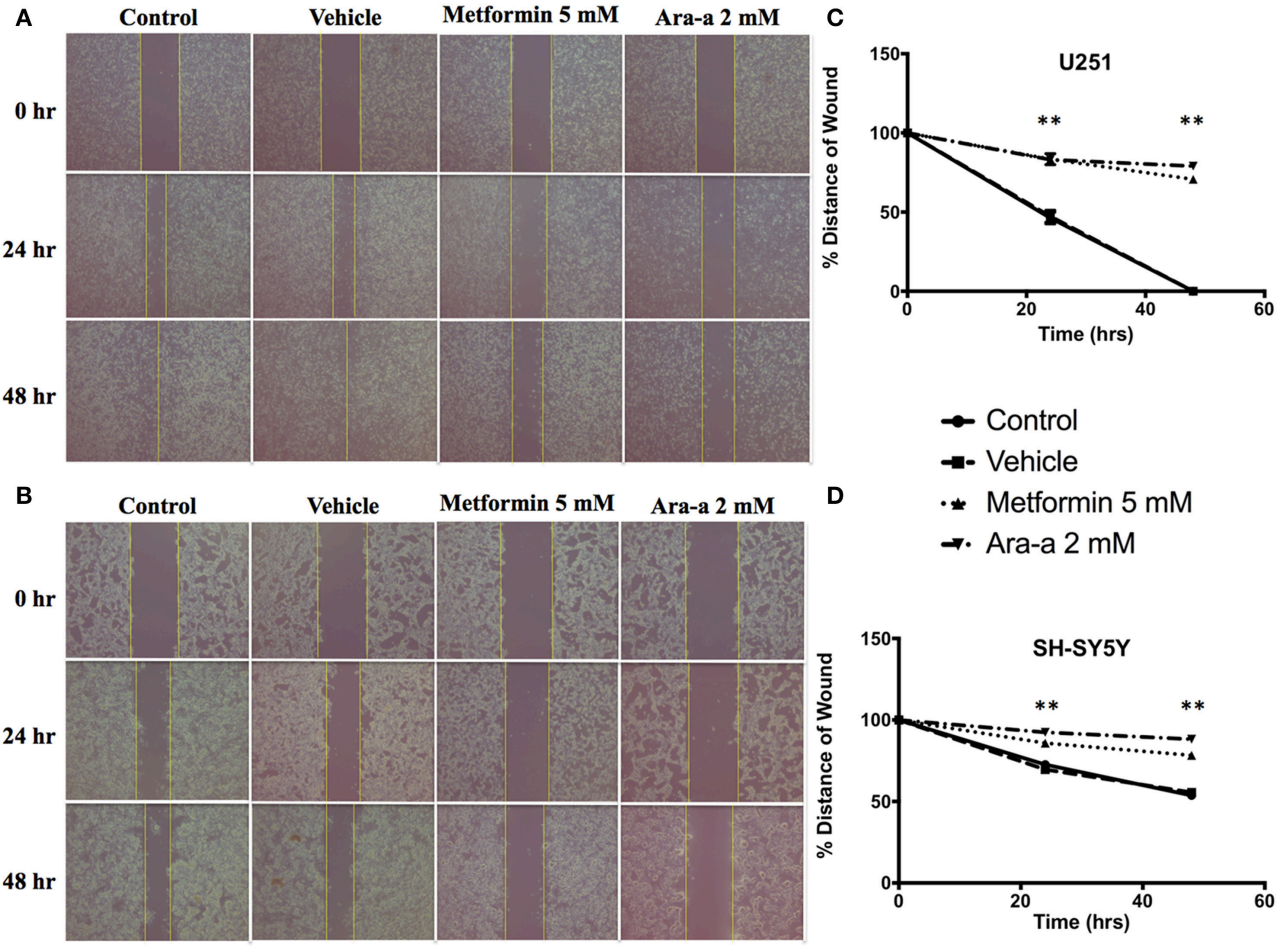
**Metformin and Ara-a reduce the migratory and invasive potentials of human neuronal and glial cancer cell lines**. A scratch was made in a six-well plate of confluent U251 cells with Mitomycin C **(A)** and SH-SY5Y cells without Mitomycin C **(B)**, using a 200 μl tip, and images were taken at *T* = 0, 24, and 48 h with or without treatment, and quantification of the distance of the wound closure was assessed over time **(C,D)**. Results are expressed as a percentage of each group compared to its condition at *T* = 0 h. Data represent an average of three independent experiments. The data are reported as mean ± SEM (^**^P < 0.01).

**Figure 3:**
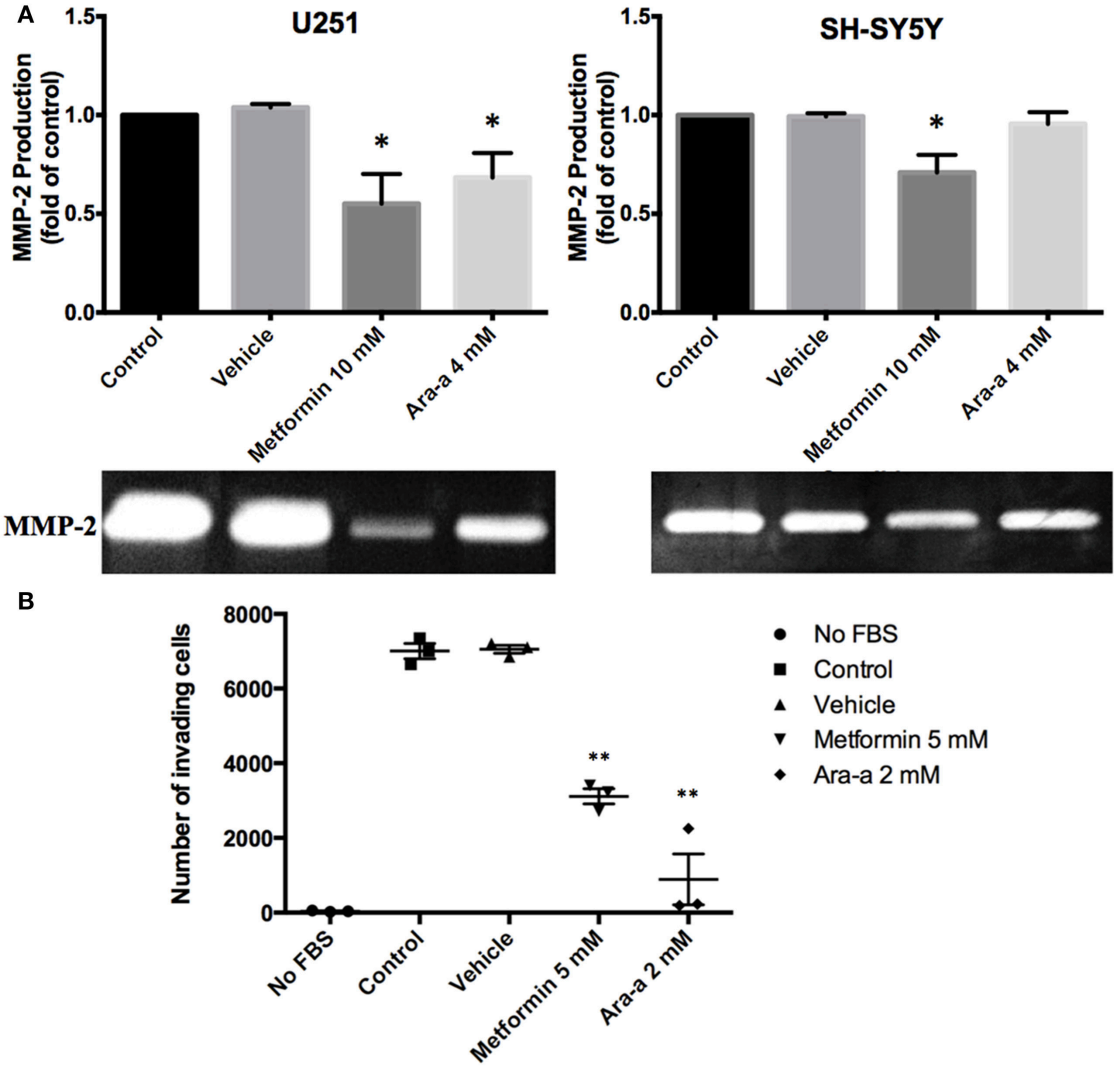
**The effect on matrix metalloproteinase-2 (MMP-2) production and invasion potential. (A)** After seeding U251 and SH-SY5Y cell lines and treating them with 10 mM of Metformin or 4 mM of Ara-a, the culture supernatant was collected and zymography was performed to detect changes in MMP-2 production following treatment, which are reflected in changes in gel digestion band size. The band size was measured via Image J software. Results are expressed as a percentage of the treated group compared to its control. Data represent an average of three independent experiments. The data are reported as mean ± SEM (^*^P < 0.05). **(B)** U251 cells were seeded onto the Matrigel™-coated membrane in the top chamber of the trans-well and were either treated or not treated with 5 mM of Metformin or 2 mM of Ara-a in the presence of FBS in the lower chamber. Cells that invaded to the lower chamber after 24 h were fixed with methanol, stained with Hematoxylin and Eosin, counted and represented as a number of invaded cells. Data represent an average of three independent experiments. The data are reported as mean ± SEM (^**^P < 0.01).

We then investigated the effect of our drugs on cell migration and invasion, which are two major pillars of cancer metastasis and, in turn, prognosis. A wound-healing assay was performed on U251 in the presence of Mitomycin C, a proliferation inhibitor. Mitomycin C was not used in the migration assay of SH-SY5Y since it led to their death. Both Metformin and Ara-a were able to significantly suppress the migration of U251 cells, while the untreated cells were able to completely close the wound at 48 h (Figures 2A,C). Even though untreated SH-SY5Y cells were not able to completely close the wound at 48 h, both drugs similarly significantly inhibited the migration of treated cells (Figures 2B,D). This shows that Metformin and Ara-a can suppress the metastatic ability of both cancer cell lines. These results were also compatible with the trans-well invasion assay on U251 cells, whereby Metformin significantly decreased invasion by around 2.5 folds while Ara-a reduced invasion even more, by around seven folds (Figure 3B). The assay was not performed on SH-SY5Y cells since they failed to show an ability to invade when using FBS as a chemo-attractant. This shows that both Metformin and Ara-a are capable of reducing the invasive ability of glioblastoma.

### Metformin and Ara-a inhibit the secretion of MMP-2

Matrix metalloproteinases are known to aid cancer cells in digesting their surrounding matrix and increasing their ability to invade through the basement membrane (Gialeli et al., 2011). This is clinically significant in the sense that MMP production by tumor cells could aid their metastasis to other sites of the body and thus make cancer treatment even more cumbersome. We sought to assess the effect of our drugs on the production of MMP-2 and MMP-9. Zymography electrophoretic technique was performed to detect changes in the production of both MMPs, following treatment. As a result, we found that only Metformin significantly decreases the production of MMP-2 in both cells lines, while Ara-a affects U251 cells only (Figure 3A). Furthermore, the effect of both drugs on MMP-2 production was more potent on U251 compared to SH-SY5Y cell lines. MMP-9 was secreted in negligible amounts by neuroblastoma and glioblastoma cell lines and both drugs had no effect on its production (data not shown).

### Sphere forming capability of U251 and SH-SY5Y

**Figure 4:**
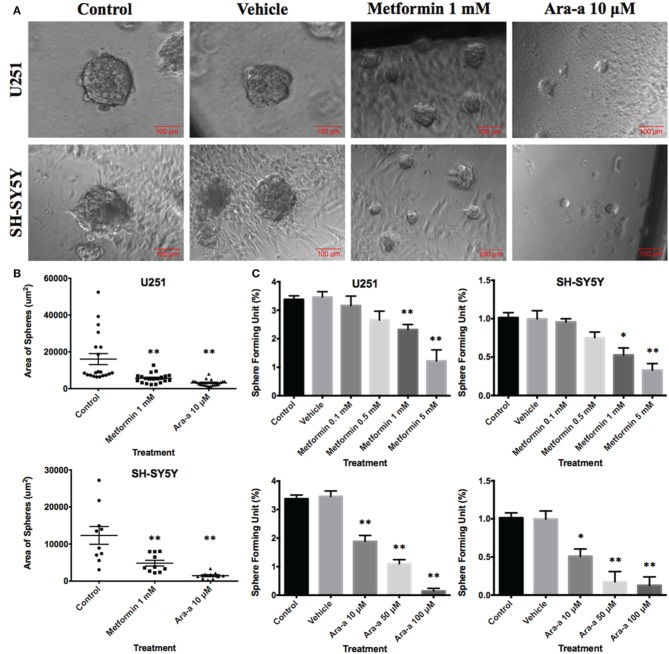
**Effect of Metformin and Ara-a on the sphere-forming ability of human neuronal and glial cancer cell lines. (A)** Representative bright-field images of U251 and SH-SY5Y neurospheres with or without Metformin and Ara-a treatment. Images were visualized by Axiovert inverted microscope at 10x magnification and analyzed by Carl Zeiss Zen 2012 image software. **(B)** Quantification of the average size of U251 and SH-SY5Y neurospheres with or without treatment conditions. Data represent an average area (μm^2^) of 20 measured U251 spheres and 10 measured SH-SY5Y spheres. The data are reported as mean ± SEM (^**^P < 0.01). **(C)** Increasing the concentration of treatment of both Metformin and Ara-a on U251 and SH-SY5Y spheres, cultured in Matrigel™, led to a similar decrease in the number of Sphere Forming Units (SFU) in both cell lines. The generated spheres are referred to as G1 (Generation 1) spheres. Results are expressed as SFU which is calculated according to the following formula: SFU = (number of spheres counted x number of input cells) × 100. Data represent an average of three independent experiments. The data are reported as mean ± SEM (^*^P < 0.05; ^**^P < 0.01).

In order to better visualize their sphere-forming capabilities, single cell suspensions of U251 and SH-SY5Y were cultured in Matrigel™ for 9 and 14 days, respectively. The spheres were then visualized under an inverted light microscope and bright-field images were acquired (Figure 4A). U251 cell line produced more and relatively larger spheres compared to those of SH-SY5Y (Figures 4A,B).

### Metformin and Ara-a target an enriched population of U251 and SH-SY5Y cancer stem/progenitor cells

Our main focus in this study is to see the potential inhibitory effects of Metformin and Ara-a on tumor survival by targeting its CSC population *in vitro*, which is translated by the ability to form and propagate spheres. Neurospheres were generated from 10,000 single cells of U251 and SH-SY5Y, embedded in Matrigel™. After culturing U251 and SH-SY5Y cells for 9 and 14 days, respectively, we got an SFU of around 3.4 and 1% for each of U251 and SH-SY5Y, respectively (Figure 4C). The number and size of cultured neurospheres decreased in a dose-dependent manner for both drugs (Figure 4C), whereby the number of spheres were significantly decreased in both cell lines at 1 and 5 mM of Metformin and at 10, 50, and 100 μM of Ara-a. Treatment with 1 mM of Metformin significantly decreased neurosphere formation by around 33% and 50% for U251 and SH-SY5Y cell lines, respectively. On the other hand, 10 μM of Ara-a significantly decreased sphere formation in both cell lines by around 50%. We noticed that the effect of Metformin and Ara-a was more pronounced on the 3D culture compared to 2D, whereby a lower concentration of each drug, specifically Ara-a, led to a stronger anti-survival effect on the cancer cells.

**Figure 5:**
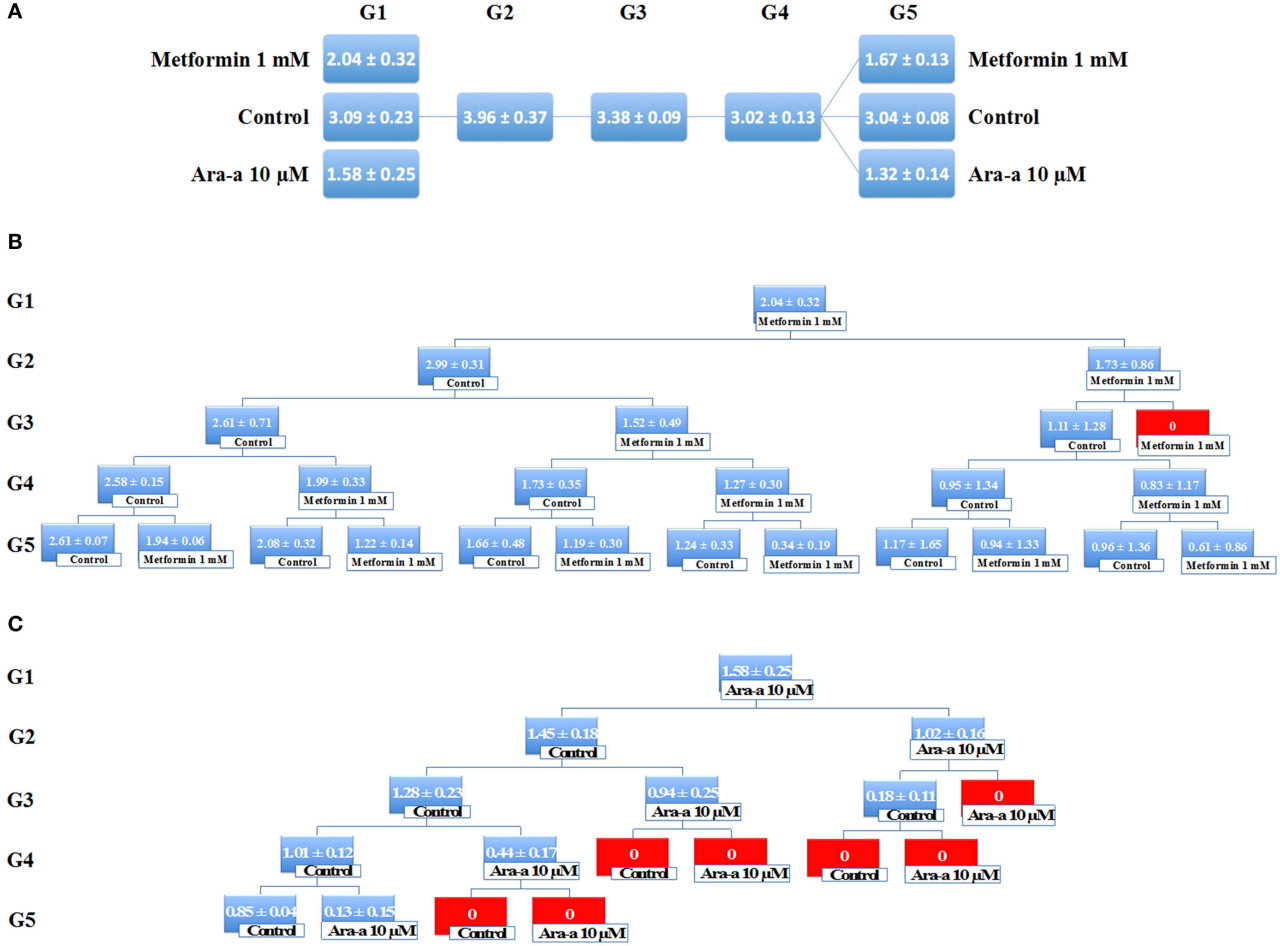
**Metformin and Ara-a inhibit the self-renewal ability of the glial cancer stem/progenitor cells. (A)** SFU obtained from serially passaged U251 neurospheres over five generations is shown under untreated conditions (always control) and with 1 mM Metformin and 10 μM Ara-a-treated condition (treated once at G1 and at G5). **(B,C)** Glial CSC were enriched from U251 cell line and treated with either Metformin (1 mM) **(B)** or Ara-a (10 μM) **(C)**. After each propagation, cells that were initially treated with Metformin, Ara-a or media (control) were seeded into separate wells. Spheres were propagated for five generations. SFU is counted and an average of two independent experiments is represented. The data are reported as mean ± SD.

Being the hallmark of stem/progenitor cells, we investigated the effect of Metformin and Ara-a on the neurosphere self-renewal activity, by assessing sphere formation capability over five generations. Since SH-SY5Y cell lines did not survive propagation, this assessment was only done on the U251 cell line, whereby U251 spheres of the first generation (G1) were collected and propagated by dissociating them into single cells and reseeding the same number of cells used initially (10^4^cell/well). The experimental design of this assay is illustrated in Figure 5. Treatment with 1 mM of Metformin led to around 33% decrease in sphere formation in G1, whereas treatment with 10 μM of Ara-a led to around 50% decrease in sphere formation in G1 (Figure 5A). After enriching for cancer stem/progenitor cells by propagating untreated cells, treatment of U251 neurospheres at G5 with either Metformin or Ara-a yielded approximately the same decrease in SFU as that witnessed when treating G1 spheres (Figure 5A). Upon successive propagation and treatment of cells, SFU progressively decreased to reach 0% at G3 after continuous Metformin treatment (Figure 5B). Ara-a was even more potent in reducing the SFU of U251 cells whereby most propagations ended in an SFU of 0% by the fourth generation, while most of the Metformin-treated spheres remained viable up till the fifth generation (Figure 5C). Interestingly, it was noted that upon withdrawal of Metformin treatment in the subsequent generation, the neurospheres were able to regain some of their sphere-forming ability, yet this was not the case with Ara-a, whereby after one Ara-a treatment, the subsequent untreated generations produced a lower SFU.

## Discussion

Cancer therapy is always evolving to become more potent and efficient in eradicating tumors with the least number of side effects on the patient. Biochemical and molecular studies of oncogenesis have investigated the contribution of various parameters involved in the pathogenesis of cancer, including the AMPK pathway (Godlewski et al., 2010; Laderoute et al., 2014; Tan et al., 2014; Kim et al., 2015; Sujobert et al., 2015). In addition to its anti-cancerous effects, activating the AMPK pathway via Metformin was shown to be protective against neuro-degeneration by inhibiting the formation of advanced glycosylation end products, which reduce the expression of neuro-protective genes (Bcl-2, CREB and PPARγ) (Chung et al., 2015). Thus, by interfering in the LKB1/AMPK/mTOR/S6K1 pathway, Metformin seems to be a promising drug in the field of neuro-oncology whereby it can destroy cancerous brain cells and their CSCs (Carmignani et al., 2014), revitalize healthy neurons, and even prevent chemotherapy-induced neuropathy (Mao-Ying et al., 2014). With the AMPK pathway proving to be a good therapeutic target, and our belief that eradicating CSCs is the hallmark for a long-term cure of cancer, we conducted this study to investigate the role of an AMPK activator and inhibitor on the survival of cancer cell lines and their CSC population.

This study showed that both drugs, Metformin and Ara-a significantly affect the proliferation and survival of cancer cells of brain origin. The fact that both drugs decrease viability yet work antagonistically on the AMPK pathway, we hypothesize that each drug may be affecting alternative pathways. One explanation is that AMPK activation leads to the inhibition of the mTOR pathway, in addition to the fact that Metformin itself inhibits the Akt (Wurth et al., 2013) and mTOR pathway (Yu et al., 2015), which is downstream of both Akt and AMPK. Both the mTOR and Akt pathways have been implicated in various types of cancers (Vivanco and Sawyers, 2002; Hsieh et al., 2015; Sharma et al., 2015b; Wang et al., 2015). On the other hand, given that the AMPK pathway directs cellular signaling into a catabolic state to produce ATP in the case of cellular energy deficiency (Viollet et al., 2010; Egan et al., 2011; Suzuki et al., 2013), Ara-a-induced inhibition of AMPK may be depriving the cancer cells from producing the energy they need to survive. Yet, Ara-a is a nucleoside analog and can interfere with DNA synthesis and may thus exert anti-cancerous effects via that mechanism which may explain the higher potency of Ara-a compared to Metformin.

Another mechanism in which these drugs were affecting these cells and inducing their death was mediated via the increase of intracellular calcium (data not shown), which prompts further investigations in future studies to elucidate the exact mechanisms mediating the anti-cancerous effects. Furthermore, both Metformin and Ara-a had an inhibitory effect on the invasive ability of both glial and neuronal cancer cells, possibly through decreasing MMP-2 production that is known to have a role in cancer progression by aiding extracellular matrix degradation. This could provide cancer patients with a great benefit in terms of increasing their survival and disease prognosis. With gliomas being extremely fatal due to their enormous proliferative and invasive capacity, these drugs could at least significantly hinder disease progression, allowing an easier brain excision with the least complications possible.

Most importantly, we studied the effect of the AMPK activator and inhibitor on the stem cell population within the cell lines. The existence of a CSC population within different types of tumors are continuously being uncovered and this population of cells have been correlated with a poorer prognosis, irrespective of the type of cancer. These included cancers of the brain, blood, ovaries, colon, breast and prostate (Asfaha et al., 2015; Duru et al., 2015; Huang et al., 2015; Kato et al., 2015; Li et al., 2015; Yin et al., 2015). Many studies are currently being conducted to phenotype these cells and understand their genetic makeup and the regulatory pathways involved in guiding their cell cycle as undifferentiated cells having progenitor stem-like properties (Fares et al., 2015; Mathur et al., 2015; Moreira et al., 2015; Sharma et al., 2015a). Unfortunately, most current anti-cancer treatments target the highly proliferative cancer cells, constituting the bulk of the tumor, while missing the slowly dividing treatment-resistant stem cells that is believed to lead to cancer recurrence after an apparent remission (Yu et al., 2012). After finishing all the cycles of cancer treatment and entering a phase of remission, these few CSCs could replenish the eradicated progenitor cells, which in turn re-establish the tumor. Thus, it is crucial to use a drug that targets this small CSC population in order to ensure the prevention of a recurrence. That is why we studied the ability of Metformin and Ara-a in depleting glioma CSCs, via assessing their effect on sphere-formation.

We found that both drugs significantly decrease the number and size of SFUs, and this effect is additive with higher concentrations and as the spheres are passaged into subsequent generations, with Ara-a being more potent and efficient than Metformin. However, we noticed that some of the sphere-forming capabilities are regained if Metformin treatment is withdrawn after passaging the spheres, but can be inhibited again with another cycle of treatment. This shows that a serial treatment with Metformin is needed to completely and efficiently destroy the CSC population and prevent tumor recurrence. On the other hand, the effect of a single Ara-a treatment on G1 could still be observed until the fifth generation of U251 spheres. It seems that Ara-a has a unique mechanism of action, unlike Metformin, that progressively decreases cell viability and sphere-forming capacity of CSCs even after the removal of the trigger (Ara-a), and this mechanism is further enhanced if the spheres are treated again. This lingering effect could be due to the fact that once the cells are exposed to Ara-a, it constantly disrupts their DNA synthesis by becoming incorporated into the CSC DNA as Ara-ATP. Noteworthy, Metformin turned out to be selectively anti-cancerous against CSCs compared to their differentiated cancer counterparts, for both U251 and SH-SY5Y. On the other hand, in addition to being potently anti-cancerous against the CSCs of U251 and SH-SY5Y, Ara-a also effectively eradicated the highly proliferative U251 cells but not the SH-SY5Y. This goes to show that with Metformin and Ara-a being effective against CSCs, an adjunct drug/therapy could be utilized to efficiently target the highly proliferative cells comprising the tumor bulk of brain cancer.

In conclusion, our study reveals that targeting the AMPK pathway, through an inducer or inhibitor, presents an effective anti-cancerous treatment whereby they target the main pillars of its fatality, namely its metastatic and regenerative properties. Optimizing the treatment of Metformin and/or Ara-a could be sufficient in eradicating brain tumors by killing the proliferating cells, inhibiting recurrence by depleting the CSC population over several generations via successive treatments, and preventing their metastasis to other organs, which ultimately increase the severity of multi-organ cancer complications. More importantly, our study showed that using an AMPK inhibitor was not only effective but even more potent than an inducer, in the treatment of brain cancer and its CSCs. This requires further investigations on AMPK inhibition to assess the differentially regulated genes that are leading to the potent effect of Ara-a on cancer cells. It is also worth assessing for the presence of the same cytotoxic effect on healthy neurons and glial cells. Future studies need to be done to further assess the effectiveness of these drugs, specifically Ara-a, on the CSCs in animal and human tumors of the brain and other organs, whereby their inclusion as adjuncts in current anti-cancer treatments could provide better therapeutic results.

## Author contributions

TM, AN, MI contributed to the project design and execution of experiments, analysis of results, and writing of manuscript. FC, HB, AM, RE contributed to execution of experiments, results analysis and manuscript writing. GD, AE, FK contributed to overlooking and following up with experiments, result analysis and manuscript proofreading. WA contributed to project design, result analysis, manuscript writing and proofreading.

## Funding

This research was supported by funding from the Medical Practice Plan (MPP) at AUB-FM. The funders had no role in study design, data collection, and analysis, decision to publish, or preparation of the manuscript.

### Conflict of interest statement

The authors declare that the research was conducted in the absence of any commercial or financial relationships that could be construed as a potential conflict of interest.
